# Depletion of the m1A writer TRMT6/TRMT61A reduces proliferation and resistance against cellular stress in bladder cancer

**DOI:** 10.3389/fonc.2023.1334112

**Published:** 2024-01-18

**Authors:** Ida Monshaugen, Luisa Luna, Jayden Rhodes, Felicia Iselin Svensson Kristiansen, Anna Lång, Stig Ove Bøe, Anindya Dutta, Zhangli Su, Arne Klungland, Rune Ougland

**Affiliations:** ^1^ Centre for Embryology and Healthy Development, Department of Microbiology, Oslo University Hospital Rikshospitalet, Oslo, Norway; ^2^ Department of Molecular Medicine, Institute of Basic Medical Sciences, University of Oslo, Oslo, Norway; ^3^ Department of Surgery, Baerum Hospital Vestre Viken Hospital Trust, Gjettum, Norway; ^4^ Department of Genetics, University of Alabama at Birmingham, Birmingham, AL, United States; ^5^ Centre for Embryology and Healthy Development, Department of Clinical Medicine, Faculty of Medicine, University of Oslo, Oslo, Norway

**Keywords:** RNA modification, bladder cancer, non-coding RNA, m1A regulators, N1-methyladenosine, TRMT6/TRMT61A

## Abstract

**Background:**

Bladder cancer (BLCA) is a common and deadly disease that results in a reduced quality of life for the patients and a significant economic burden on society. A better understanding of tumorigenesis is needed to improve clinical outcomes. Recent evidence places the RNA modification m1A and its regulatory proteins TRMT6/TRMT61A and ALKBH3 in BLCA pathogenesis.

**Methods:**

TRMT6/TRMT61A, ALKBH1, and ALKBH3 expression was examined in human BLCA cell lines and a normal urinary tract epithelium cell line through qRT-PCR and western blot analysis. Prestoblue Cell Viability Reagent, wound-healing assay, and live-cell imaging-based cell displacement analysis, were conducted to assess proliferation, migration, and displacement of this BLCA cell line panel. Cell survival was assessed after inducing cellular stress and activating the unfolded protein response (UPR) with tunicamycin. Moreover, siRNA-mediated gene silencing in two BLCA cell lines (5637 and HT1197) was conducted to investigate the biological roles of TRMT6/TRMT61A.

**Results:**

Heterogeneous morphology, proliferation, displacement, tunicamycin sensitivity, and expression levels of m1A regulators were observed among the panel of cell lines examined. In general, TRMT61A expression was increased in BLCA cell lines when compared to SV-HUC-1. Depletion of TRMT6/TRMT61A reduced proliferation capacity in both 5637 and HT1197 cell lines. The average cell displacement of 5637 was also reduced upon TRMT6/TRMT61A depletion. Interestingly, TRMT6/TRMT61A depletion decreased mRNA expression of targets associated with the ATF6-branch of the UPR in 5637 but not in HT1197. Moreover, cell survival after induction of cellular stress was compromised after TRMT6/TRMT61A knockdown in 5637 but not in HT1197 cells.

**Conclusion:**

The findings suggest that TRMT6/TRMT61A plays an oncogenic role in BLCA and is involved in desensitizing BLCA cells against cellular stress. Further investigation into the regulation of TRMT6/TRMT61A expression and its impact on cellular stress tolerance may provide insights for future BLCA treatment.

## 1 Introduction

Bladder cancer (BLCA) is the 10^th^ most frequently diagnosed cancer worldwide, accounting for 573,000 new annual diagnoses and 213,000 deaths. Due to gender discrepancy, BLCA is the 6^th^ most common cancer in men and the 9^th^ leading cause of cancer death (1). Meanwhile, there is an increasing incidence in women. Thus, BLCA poses a significant public health concern at a global level.

The most frequent type of BLCA is non-muscle invasive bladder cancer (NMIBC), accounting for about 75% of newly diagnosed cases (2). Despite being characterized by a favorable prognosis and overall survival, relapses are frequent, leading to lifelong control schemes, costly treatment, and poor quality of life. Patients with muscle-invasive bladder cancer (MIBC) face poor prognoses and challenges related to major surgery and urinary diversions as well as chemo- and radiotherapy resistance and risk of metastasis (3). The treatment scheme for NMIBC is transurethral resection of the bladder tumor (TURBT), often combined with intravesical instillation of Bacillus Calmette-Guérin (BCG). For MIBC, radical cystectomy combined with urinary diversion is currently the primary treatment strategy (4). However, treatment strategies for BLCA are advancing. For NMIBC, this includes strategies to improve the delivery and uptake of current therapeutic agents and the development of new immunotherapies. For MIBC, immune checkpoint inhibitors (ICIs) are promising candidates in maintenance therapy. For more details on advances in BLCA therapeutic strategies, the reader is referred to excellent reviews (5, 6).

N1-methyladenosine (m1A) is an important post-transcriptional modification and occurs throughout the tRNA structure (7). m1A at position 58 (m1A58) is the predominant m1A modification of cytoplasmic tRNA in eukaryotic cells (8). The formation of m1A58 on cytoplasmic tRNAs is mediated by the methyltransferase complex TRMT6/TRMT61A, in which TRMT61A functions as the catalytic subunit and TRMT6 is responsible for tRNA binding (9). Members of the AlkB protein family, ALKBH1 and ALKBH3, can catalyze m1A removal in tRNAs by oxidative demethylation (10–12). Dysregulation of TRMT6/TRMT61A has been reported in various cancers (13–15). In BLCA, increased hTrm6p/hTrm61p expression and elevated m1A levels promote disease progression (16). Recently, we reported increased TRMT6/TRMT61A expression accompanied by elevated m1A levels on a tRF class in BLCA and suggested that this promotes the unfolded protein response (UPR), most likely through regulation of m1A base modification on these tRFs (17). UPR is a cellular response activated when cells face an imbalance in the cellular environment, resulting in the accumulation of misfolded or unfolded proteins due to intrinsic or extrinsic stressors. Emerging evidence suggests that by enabling cancer cells to cope with surrounding stress stimuli, the UPR is a central player in malignant transformation and tumorigenesis by regulating cancer cell survival, angiogenesis, metastasis, and chemoresistance (18). Moreover, elevated ALKBH3 expression is found in several cancers, including BLCA (19). Thus, evidence suggests that dysregulation of m1A regulators is involved in BLCA tumorigenesis, but the underlying mechanisms remain elusive.

In this study, a panel of BLCA cell lines and a bladder epithelial control cell line were characterized with regard to expression levels of the m1A methyltransferase complex TRMT6/TRMT61A and demethylases ALKBH1 and ALKBH3. The biological role of TRMT6/TRMT61A on proliferation, migration, and displacement was investigated in two BLCA cell lines. Moreover, the effect of TRMT6/TRMT61A on UPR activation and stress tolerance was further investigated in these two BLCA cell lines.

## 2 Methods and materials

### 2.3 Cell culture

All cell lines were obtained from the American Type Culture Collection (ATCC, Manassas, VA, USA). SV-HUC-1 (male) (#CRL-9520) cells were maintained in F-12K medium (ATCC #30-2004), 5637 (male) (#HTB-9) cells in RPMI 1640 (VWR, #L0498), T24 (female) (#HTB-4) cells in McCoy’s 5a MM (ATCC, #30-2007), SW780 (female) (#CRL-2169) in DMEM (Merck life science, #D6429-24X500ML) and HT1197 (male) (#CRL-1473) and HT1376 (female) (#CRL-1472) cells in EMEM (#30-2003). All media was supplemented with 10% fetal bovine serum (Merck life science, #F7524) and 100 U/ml Penicillin-Streptomycin (Gibco, #15140130). Cells were cultured in a humidified atmosphere with 5% CO_2_ at 37°C.

### 2.2 Transfection with siRNA

Small interfering RNAs (siRNAs) targeting TRMT6 (Horizon Dharmacon, #L-017324-02-0005), TRMT61A (Horizon Dharmacon, #L-015870-01-0005), and negative control (Horizon Dharmacon, #D-001810-10-20) were transfected into cancer cells with Lipofectamine™ RNAiMAX Transfection Reagent (Invitrogen, #13778150) in OptiMEM™ I Reduced Serum Medium (Fisher Scientific, #31985070) at a 10nM final siRNA concentration. Cells were transfected twice and collected 96 h post first transfection (48 h post second transfection).

### 2.3 Cell proliferation and survival assay

PrestoBlue™ Cell Viability Reagent (A13262, Invitrogen) was utilized for cell viability, and this assay was carried out according to the manufacturer’s instructions. Briefly, cells were seeded onto 96-well plates (VWR/Falcon, 353072). Cell proliferation and survival (refer to Section 2.4 Tunicamycin treatment) were assessed by adding 10 µL Prestoblue (Invitrogen, A13262) to each well containing 100 µL medium. After incubating for 10 minutes (15) at 37°C, fluorescence was measured on a plate reader (VICTOR Nivo Multimode plate reader, Perkin Elmer). Between three and five independent experiments were carried out to measure proliferation and survival after tunicamycin treatment.

### 2.4 Tunicamycin treatment

For cell survival after tunicamycin (Merck/Sigma-Aldrich, #T7765) treatment, approximately 6000 cells were plated in 96 well plates. After 4 hours (h), tunicamycin was added to the wells at different concentrations (100, 200, 400, 600 and 800 nM), and cells were allowed to grow for 76 h. Cell viability was analyzed at four timepoints: 4 h after seeding (at the time tunicamycin was added, T=0) and 24, 48 and 72 h (T=24, T=48 and T=72) after tunicamycin addition. To induce ER stress and subsequently activate the UPR, cells were treated with 5 µg/mL tunicamycin for 8 h.

### 2.5 RNA isolation

Total RNA of bladder cancer cell lines was extracted using RNAzol® RT reagent (Molecular Research Center, #MR-RN190-500) according to the manufacturer’s instructions. RNA quality and quantity was assessed with Nanodrop (Thermo Scientific™ NanoDrop™ One Microvolume UV-Vis Spectrophotometer).

### 2.6 Reverse transcription-quantitative PCR (RT-qPCR assays)

cDNA was obtained using SuperScript™ IV VILO™ Master Mix with ezDNase™ Enzyme (Invitrogen, #11766050) according to manufacturer’s instructions. 500 ng total RNA (refer to Section 2.5 RNA isolation) was used per cDNA reaction. For mRNA analysis, qRT-PCR was performed with PowerUp™ SYBR™ Green Master Mix (Applied Biosystems; Thermo Fisher Scientific, USA, #A25742) using the StepOne Plus and QuantStudio™ 5 Real-Time PCR Systems (Applied Biosystems; Thermo Fisher Scientific, USA, #A28140). 4 ng cDNA was used in 10 µL reaction. All sequences for primers used are listed in **Supplementary Table 1**. Fold changes in mRNA expression were determined using the 2^-ΔΔCT^ method and normalized against GAPDH or ACTB as the endogenous control and further normalized relative to SV-HUC1 with StepOne™ Software v2.3.

### 2.7 Western blotting

Protein was extracted from cell pellets (trypsinized and washed in PBS and kept at -20C prior to use) using RIPA Lysis and Extraction Buffer (Thermo Scientific™ #89900) with Protease Inhibitor Cocktail (Merck Life Sciences #O8340-5ML) added to 1X. Protein concentration was estimated by Bradford Assay (BioRad #500-0006). For Western blot analysis, 20 μg protein lysate was mixed with 4X Bolt™ LDS Sample Buffer (Invitrogen #B0007), heated for 10 min at 70°C and separated on Bolt 12% Bis-Tris Plus gel (Invitrogen #NW00120BOX) for 1.5 h at 150V. Proteins were transferred onto a PVDF membrane (Biorad #1704156) with a Trans-blot turbo system (Biorad). Following blocking of the membranes for 1 h in 5% milk in PBS with 0.05% Tween-20 (PBST), the primary antibodies and corresponding dilutions were used in 5% milk in 0.05% PBST: anti-GAPDH (Abcam, #ab125247, mouse monoclonal, used at 1:3000), anti-TRMT6 (Abcam #ab235321, rabbit polyclonal, used at 1:1000), anti-TRMT61A (Biorbyt #orb411814, rabbit polyclonal, used at 1:500), anti-ALKBH1 (Abcam #ab128895, rabbit monoclonal, used at 1:3000), anti-ALKBH3 (Cell Signaling Technology #87620, rabbit monoclonal, used at 1:1000). Anti-mouse (BioNordica #PI-2000, used at 1:8000) and anti-rabbit (GE Healthcare Life Sciences #NA934-100UL, used at 1:10.000) HRP-linked secondary antibodies were used for detection of the respective targets. Blots were developed using the SuperSignal West Dura Extended Duration Substrate (Thermo Fisher #34075) for GAPDH and SuperSignal West Femto Maximum Sensitivity Substrate (Thermo Fisher #34095) for TRMT6, TRMT61A, ALKBH1, and ALKBH3 on Biorad ChemiDoc XRS+ System. Band signal intensities were obtained with ImageLab Software (v5.2.1) and used to determine relative target protein levels normalized to GAPDH and further normalized to SV-HUC1, which were visualized with GraphPad Prism (v9.1.0).

### 2.8 Cell migration

For morphology analysis, 5 x 10^5^ cells were seeded in 6 well plates, and pictures were taken after 24-36 hours with an Olympus CKX53 inverted microscope. For the scratch wound healing assay, 2 x 10^6^ cells were seeded in 24-well plates. After 24 h, a pipette tip (1000 μl) was used to create a scratch wound. Next, the cell cultures were washed using PBS and incubated at 37C and 5% CO_2_. The wounds were observed under an Olympus CKX53 inverted microscope for 24 h *in situ* to compare the number of cells that migrated into the wound areas.

### 2.9 Analysis of cell displacements

Urinary bladder cancer cell lines (SV-HUC-1, 5637, SW780, T24, 5HT1376, HT1197) were seeded at sub-confluent densities (4000 cells/well) in 96-well glass bottom plates (Greiner Sensoplate, M4187-16EA, Merck). After seeding, the plates were placed in a CO_2_ incubator overnight to allow cells to attach to the glass surface. Subsequently, cells were monitored using an ImageXpress Micro Confocal high-content microscope (Molecular Devices), equipped with an environmental chamber maintaining 5% CO_2_ and 37°C, and controlled by the MetaXpress 6 software. Time lapse series of phase contrast images were acquired in widefield mode using a 20x 0.45 NA Ph1 air objective, camera binning = 2, a time interval of 3 min between frames and a total imaging period of 20 h. Cell displacement were analyzed by particle tracking using the TrackMate plugin in Fiji ImageJ (20) in combination with *in house* Python-based script (Python 3.7.6). Average displacement speed was calculated as the average of all tracked cell displacements within a 12 h period of imaging.

### 2.10 Cell displacement analysis after siRNA knockdown

5637 cells treated with siRNAs were seeded at a density of 50000 cells/well in 96-well glass bottom plates (Greiner Sensoplate, M4187-16EA, Merck) and placed in a CO_2_ incubator overnight. Prior to imaging, cells were stained with NucBlue solution (Invitrogen) to label the nuclei of live cells. In brief, cells were incubated at 37°C and 5% CO_2_ for 30 min with NucBlue solution diluted 1:40 in culture medium. Subsequently, cells were washed twice with pre-warmed culture medium. Image acquisition was carried out in an ImageXpress Micro Confocal high-content microscope (Molecular Devices) in widefield mode. Time lapse series were acquired for a total time period of 4.5 h using a time interval of 4 min between frames, a 20x 0.45 NA Ph1 air objective, filter set for detection of DAPI and phase contrast, and an environmental control gasket that maintain 37°C and 5% CO_2_. Cell displacement was analyzed by particle tracking using the TrackMate plugin in Fiji ImageJ (20) in combination with *in-house* Python-based script (Python 3.7.6). Average displacement speed was calculated as the average of all tracked cell displacements within a 4 h period of imaging.

### 2.11 Quantification of RNA modifications using LC-MS/MS

Cells were harvested and stored in RNAlater™ solution (Invitrogen, #AM7021) prior to total RNA isolation (refer to Section 2.5 RNA isolation). tRNAs were enriched using an Agilent 1260 Infinity II Analytical-Scale LC-UV Purification System with a Bio SEC-3 300 Å, 2.1 x 300 mm column (Agilent Technologies) chromatographed isocratically with 100 mM ammonium acetate pH 7 at 0.280 ml/min and 40°C for 20 min. Chromatograms were recorded at 260 nm and tRNAs were collected and lyophilized and solved in 30 μl of water. The RNA was enzymatically hydrolyzed to ribonucleosides by 20 U benzonase (Santa Cruz Biotech) and 0.2 U nuclease P1 (Sigma) in 10 mM ammonium acetate pH 6.0 and 1 mM magnesium chloride at 40°C for 1 h, then added ammonium bicarbonate to 50 mM, 0.002 U phoshodiesterase I and 0.1 U alkaline phosphatase (Sigma) and incubated further at 37°C for 1 h. The hydrolysates were added 3 volumes of acetonitrile and centrifuged (16,000 g, 20 min, 4°C). The supernatants were lyophilized and dissolved in 50 µl water for LC-MS/MS analysis of modified and canonical ribonucleosides. Chromatographic separation was performed using an Agilent 1290 Infinity II UHPLC system with an ZORBAX RRHD Eclipse Plus C18 150 x 2.1 mm ID (1.8 μm) column protected with an ZORBAX RRHD Eclipse Plus C18 5 x 2.1 mm ID (1.8 µm) guard column (Agilent). The mobile phase consisted of water and methanol (both added 0.1% formic acid) run at 0.23 ml/min, for modifications starting with 5% methanol for 0.5 min followed by a 2.5 min gradient of 5-15% methanol, a 3 min gradient of 15-95% methanol and 4 min re-equilibration with 5% methanol. A portion of each sample was diluted for the analysis of unmodified ribonucleosides which was chromatographed isocratically with 20% methanol. Mass spectrometric detection was performed using an Agilent 6495 Triple Quadrupole system with electrospray ionization, monitoring the mass transitions 268.1-136.1 (A), 284.1-152.1 (G), 244.1-112.1 (C), 245.1-113.1 (U), 282.1-150.1 (m1A), 296.1-164.1 (m^6,6^A), 413.1-281.1 (t^6^A), 285.1-153.1 (d_3_-m^6^A), 273.1-136.1 (^13^C_5_-A), and 246.1-114.1 (d_2_-C) in positive ionization mode.

### 2.12 Small RNA-seq by TGIRT and data analysis

Cells were treated with 5 µg/mL tunicamycin for 8 h to induce ER stress, and total RNA was subsequently extracted from the samples (refer to Section 2.5 RNA isolation). Small RNA-seq libraries were prepared and analyzed similar to previously (17). 500 ng purified total RNA was used as starting material and followed with NEBNext Small RNA Library Prep Set for Illumina (NEB #E7330) with slight modifications: 20-fold diluted adaptors were used, TGIRT-III (InGex #TGIRT50) was used for reverse transcription before final PCR amplification for 15 cycles. PAGE gel purified libraries were pooled and sequenced at UAB Genomics Core Lab with 100-base single-end by Illumina NovaSeq. For data analysis, cutadapt v3.4 (21) was used to trim 3’ adaptor sequence and discard trimmed reads that are shorter than 15 nucleotides or contain 5’ adaptor sequence. To map small RNAs to human reference RNA sequences, unitas v1.7.3 (22) was used with setting that allows 1 internal mismatch. Reads mapped to tRFs were grouped by parental tRNA amino acid groups and tRF types. Mismatch index (0-100%) is calculated for each nucleotide on tRFs by dividing summed mismatch to summed read coverage. To plot mismatch index across different conditions, mismatch index at the indicated position is scaled across samples and represented as heatplot in Made4 R package (23). For differential analysis, microRNA and tRF counts (at least 10 read counts) were considered as input for DESeq2 wald test (24). DESeq2 results were visualized by EnhancedVolcano R package (https://github.com/kevinblighe/EnhancedVolcano).

### 2.13 Statistical analysis

Statistical analysis was carried out using the software GraphPad Prism 10.1.1 For comparisons across different cell lines the two-tailed unequal variances Welch’s t-test was applied. When comparing measurements with the same cell line, the two-tailed student’s t-test was used. IC50 was determined by regression built in analysis non-linear regression equation [inhibitor] vs. normalized response. A two-way ANOVA test with Dunnets multiple comparisons test with a single pooled variance was used for comparing each cell lines survival against the SV-HUC-1 control line for different levels of tunicamycin.

## 3 Results

### 3.1 Expansion of urothelial cell lines

**Figure 1:**
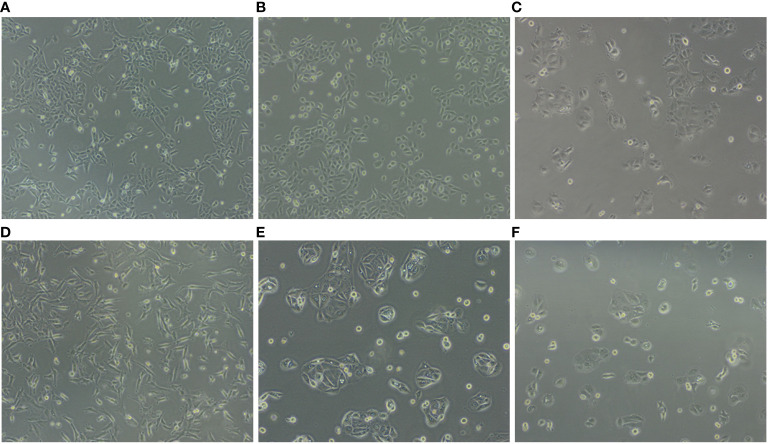
Representation of cell lines in culture highlights different features. **(A)** SV-HUC1, **(B)** 5637, **(C)** SW780, **(D)** T24, **(E)** HT1197 and **(F)** HT1376.

**Figure 2:**
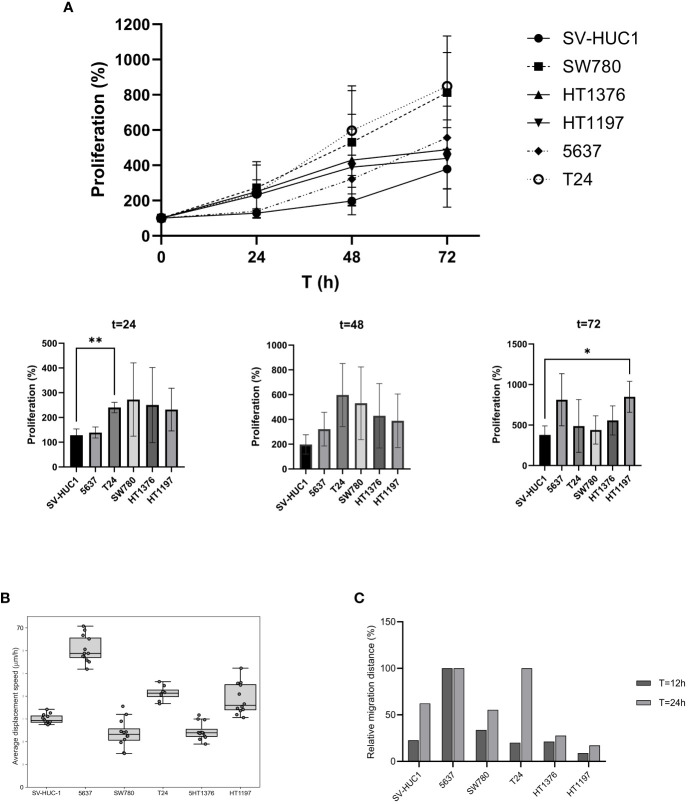
Characterization of cell proliferation, migration, and displacement. **(A)** Cell proliferation of BLCA and control cell lines grown under preferred cell culture conditions. Proliferation was compared for t = 24-, 48- and 72-hours using Welch’s t-test, two tailed. Proliferation was significantly higher for T24 at t=24 (** p = 0.016) and HT1197 at t=72 (* p = 0.0317) **(B)** Cell displacement analysis. Average displacement speed was calculated based on particle tracking data, each well contained 4000 cells. The boxplot represents the average cell displacement speed over a time period of 12 h, n = 8-12 separate microscopic fields of view. **(C)** Wound healing assay to assess cell invasiveness. Quantitation of cell migration (%) at T=12 and T=24 hours after the wound was made.

SV-HUC-1 cells derived from normal human urinary tract epithelium and five human bladder cancer cell lines (5637, T24, SW780, HT1376, and HT1197) were expanded *in vitro* in their preferred culture medium (**Table 1**) . The cultured cells presented quite different features. Upon seeding, SV-HUC-1 (**Figure 1A**), 5637 (**Figure 1B**), and T24 (**Figure 1D**) attached as individual cells. In contrast, SW780 (**Figure 1C**) and HT1376 (**Figure 1F**) formed wide clusters, while HT1197 (**Figure 1E**) formed more compact clusters. All cells proliferated to yield confluent cultures. Cell proliferation of all cell lines was examined by using the PrestoBlue Cell Viability Reagent (**Figure 2A**). The results showed the slowest proliferation for SV-HUC-1 cells compared to the rest of the bladder carcinoma cell lines, with cell lines T24 and SW780 cells proliferating the fastest. There was no correlation between the grade of the cancer cell lines and proliferation.

**Table 1:** Information overview of normal human urinary tract epithelium and five human bladder cancer cell lines used in this study.

Cell line	Gender	Age	Stage	Grade
SV-HUC1	Male	11	–	–
SW780	Female	80	Stage 1	LG
5637	Male	68	Stage 2	LG
T24	Female	81	Stage 3	HG
HT1376	Female	58	Stage 3 > pT2	HG
HT1197	Male	44	Stage 4, pT2	HG

### 3.2 Cell displacement and migration

Cell migration is a key feature of numerous biological processes such as embryo development, tissue formation, immune defense, inflammation, and cancer progression. We used two different approaches to measure cell migration in the six cell lines. First, the average cell displacement speed was investigated by time-lapse imaging for the cell lines in their preferred media (**Figure 2B**). Five of the cell lines displayed similar average displacement speed. Interestingly, one cell line, 5637, displayed a remarkably higher average displacement speed. Second, a wound-healing assay was performed, and the results confirmed the previous observations (**Figure 2C**). After 12 hours, 5637 cells had migrated across the wound.

### 3.3 Cell survival after tunicamycin treatment

**Figure 3:**
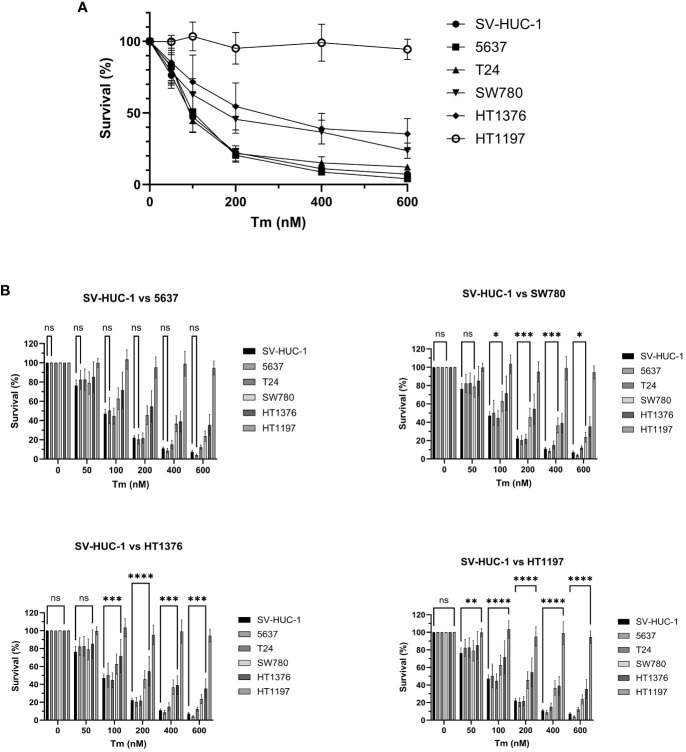
Cell survival after cellular stress and UPR induction. **(A)** Survival of BLCA and control cell lines (%) 72 h after treatment with increasing concentration of tunicamycin. The cell lines showed varying degrees of dosage sensitivity to tunicamycin. The control cell line SV-HUC1 and BLCA cell lines 5637 and T24 displayed the highest sensitivity, followed by SW780, HT1376, and HT1197. **(B)** The cell lines survival was compared to the SV-HUC-1 control line by 2-way ANOVA. The results are presented for cell lines 5637, SW780 (100 nM: p* 0.0378, 200 nM: p*** 0.0006, 400 nM: p*** 0.0002, 600 nM p*0.0255), HT1376 (100 nM: p*** 0.0009, 200 nM: p**** <0.0001, 400 nM: p*** 0.0001, 600 nM p**** 0.0001), HT1197 (50 nM p** 0.016, 100 nM: p**** <0.0001 200 nM: p**** <0.0001, 400 nM: p**** <0.0001, 600 nM p****<0.0001), ns, non-significant.

In BLCA, ER stress and the UPR have been implicated in affecting various cellular processes, including cell proliferation, apoptosis, and therapy resistance (25). Tunicamycin induces ER stress by inhibiting N-linked glycosylation, and we wanted to explore its effect on the six cell lines. Thus, in the next set of experiments, cells were treated with increasing concentrations of tunicamycin (0, 50, 100, 200, 400, 600 nM) to induce cellular stress and subsequent activation of the UPR. Subsequently, cell survival was investigated 72 hours after treatment (**Figure 3**).

The cell lines showed varying degrees of dosage sensitivity to tunicamycin, and the specific IC50 values were determined by non-linear regression (**Supplementary Figure 3**). To assess the relative sensitivity of each cell line to tunicamycin and compare it to the control cell line, we conducted a two-way ANOVA analysis (**Figure 3**). The results of the ANOVA, displayed in **Table 2**, demonstrate a significant realtionship between tunicamycin concentration, cell line and cell survival (**** p < 0.001). This indicates that the tunicamycin sensitivity varies depending on the cell line. The control cell line and four BLCA cell lines reflected a dosage-dependent tunicamycin tolerance, with SV-HUC1, 5637, and T24 having the highest sensitivity, followed by SW780 and HT1376. However, only the grade 4 cell line HT1197 survival was relatively unaffected, even with exposure to high dosages of the drug.

**Table 2:** ANOVA 2-way summary for comparing tunicamycin sensitivity in BLCA cell lines.

2-Way ANOVA for Tm sensitivity			
Source of Variation	% of total variation	P value	P value summary
Interaction	10.91	<0.0001	****
Tm (nM)	53.18	<0.0001	****
Cell line	27.07	<0.0001	****

### 3.4 Expression levels of m1A regulators in urothelial carcinoma cell lines

**Figure 4:**
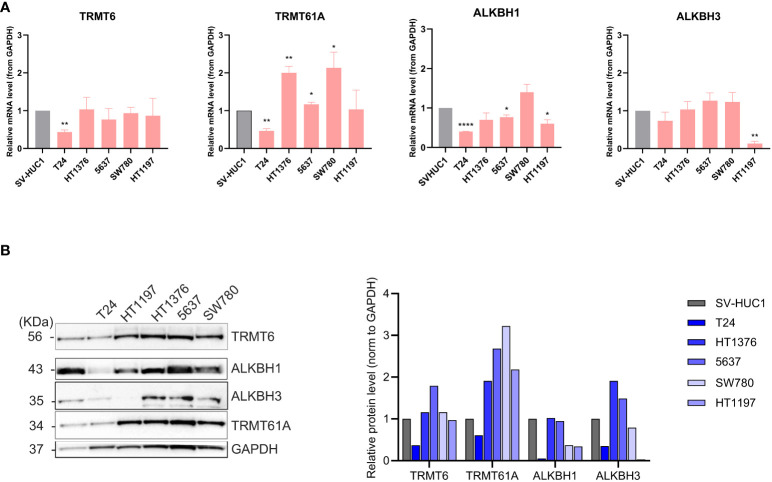
Characterization of expression levels of m1A regulators in BLCA and control cell lines. **(A)** qRT-PCR was done to check the relative mRNA expression levels of TRMT6, TRMT61A, ALKBH1, and ALKBH3. GAPDH was used as endogenous control, and mRNA expression was normalized to SV-HUC1. qRT-PCR data represented as mean and SD, based on three independent experiments. Welch’s t-test, two tailed, was used to determine statistically significant differences compared to SV-HUC. TRMT6: T24 (** p = 0.003) TRMT61A: T24 (** p = 0.004), HT1376 (** p = 0.01), 5637 (p = * 0.038), SW780 (* p = 0.042). ALKBH1: T24 (**** p = <0.0001), 5637 (* p = 0.002) HT1197 (* p = 0.02, ALKBH3: HT1197 (** p= 0.002) **(B)** Protein expression of TRMT6, TRMT61A, ALKBH1, and ALKBH3 by western blot in BLCA cell lines versus SV-HUC1 control cell line, with GAPDH as loading control. Relative protein expression was normalized to GAPDH.

The expression of m1A regulators has previously been investigated in various cancers, and upregulation of TRMT6, TRMT61A, and ALKBH3 has been reported in BLCA. We wanted to further investigate the expression levels of m1A methyltransferase subunits TRMT6 and TRMT61A and m1A demethylases ALKBH1 and ALKBH3 at the mRNA (**Figure 4A**) and protein level (**Figure 4B**). To our knowledge, this is the first time ALKBH1 expression has been investigated in BLCA. Noteworthy, T24 generally had low expression of all m1A regulators both at the mRNA and protein levels compared to SV-HUC-1. On the other hand, TRMT61A was highly overexpressed at the protein level in all the other BLCA cell lines. Compared to SV-HUC1, upregulated TRMT6 protein expression was observed at moderate levels in HT1376 and SW780 and, more prominently, in 5637. ALKBH1 protein expression levels were comparable to SV-HUC1 for HT1376 and 5637 and downregulated in HT1197 and SW780. ALKBH3 mRNA and protein expression were remarkably low in HT1197. In contrast, HT1376 and 5637 had higher ALKBH3 protein expression than SV-HUC1. In conclusion, there did not seem to be a correlation between expression levels and the stage or grade of the cancer cell lines. Furthermore, TRMT61A and ALKBH3 were upregulated in almost all BLCA cell lines compared to the control SV-HUC-1.

### 3.5 TRMT6/TRMT61A silencing inhibits cell proliferation

**Figure 5:**
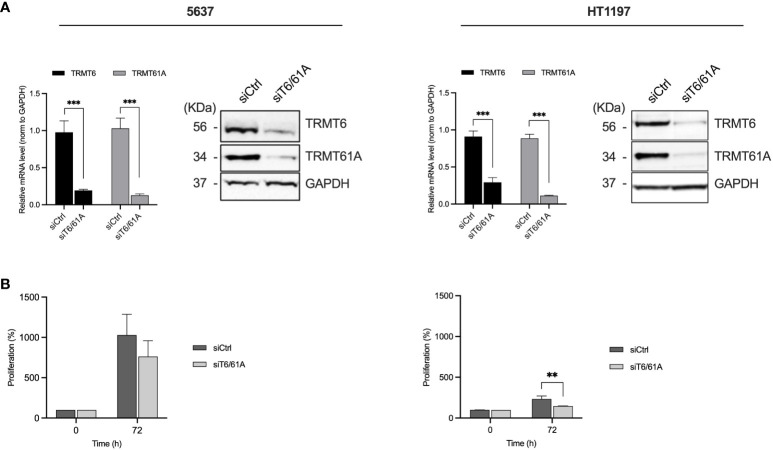
Effect on TRMT6 and TRMT61A in BLCA cell proliferation *in vitro*. **(A)** qRT-PCR and western blot were used to determine the knockdown efficiency of TRMT6 and TRMT61A in 5637 and HT1197 cell lines. Bar graphs show relative mRNA levels represented as mean ± SD, based on three independent knockdown experiments. WB shows a representative experiment for each cell line. **(B)** Cell proliferation. Knockdown of TRMT6/TRMT61A reduced cell proliferation in 5637 and HT1197. ** p-value = 0.0143, Unpaired T-test, two-tailed.

We further explored the role of TRMT6/TRMT61A in 5637 and HT1197 cells to investigate the contribution of the m1A methyltransferase complex. These cell lines were chosen based on the observed discrepancies in tunicamycin sensitivity and the malignant behavior of the BLCA cells. Moreover, both cell lines had high TRMT6/TRMT61A protein expression. The cells were transfected with siRNA pools targeting TRMT6 and TRMT61A to decrease the expression of these targets. The mRNA and protein expression levels of TRMT6 and TRMT61A were assessed by qRT-PCR and western blot, respectively (**Figure 5A**). We subsequently examined the cell proliferation of the two cell lines by the PrestoBlue Cell viability for 72 h to assess the effect of TRMT6/TRMT61A knockdown on cell proliferation (**Figure 5B**). Downregulation of TRMT6/TRMT61A reduced cell proliferation in 5637, and a significant inhibitory effect was detected in HT1197. The effect of TRMT6/TRMT61A depletion on the total m1A levels in total tRNA was quantitated by liquid chromatography-tandem mass spectrometry (LC-MS/MS) in TRMT6/TRMT61A knockdown and control samples (**Supplementary Figure 1**). A modest decrease in m1A level was detected in the 5637 cell line after TRMT6/TRMT61A depletion. However, no noticeable difference was observed for HT1197.

### 3.6 TRMT6/TRMT61A knockdown inhibits BLCA cell migration *in vitro*

**Figure 6:**
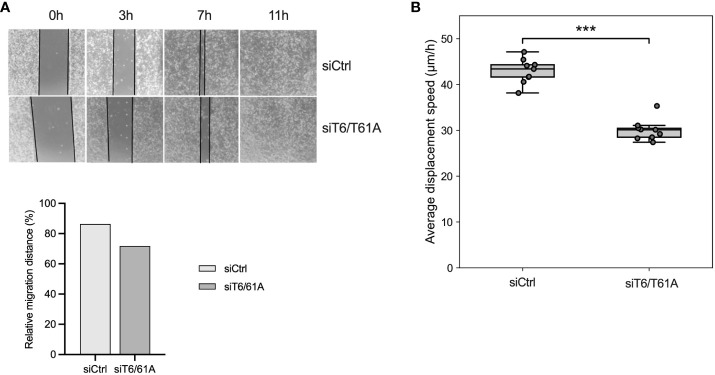
Cell migration and displacement of 5637 are inhibited by the knockdown of TRMT6/TRMT61A. **(A)**Wound-healing assay. Bars represent a relative quantitation of migration distance (%) at 7 hours after the scratch was made. **(B)** Cell displacement analysis. Average displacement speed was calculated based on particle tracking data. The boxplot represents the average cell displacement speed over a time period of 4 hours, n = 9 separate microscopic fields of view. *** p-value = 8.56·10^-9^.

To assess the role of TRMT6/TRMT61A on cell migration, a wound-healing assay was performed after TRMT6/TRMT61A knockdown for 72 hours. For 5637, siTRMT6/TRMT61A cells showed a reduced migration capacity, although not statistically significant (**Figure 6A**). However, cell displacement analysis revealed that siTRMT6/TRMT61A cells had a significantly lower average displacement speed as compared to the control (**Figure 6B**).

### 3.7 TRMT6/TRMT61A knockdown influences the UPR and sensitizes cells to tunicamycin

**Figure 7:**
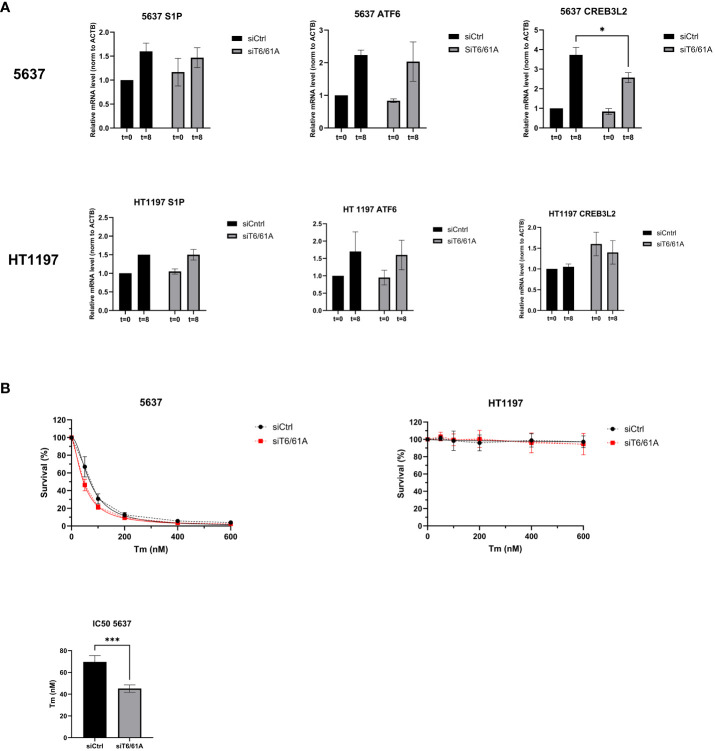
The Unfolded Protein Response and cell survival. **(A)** 5637 and HT1197 cells were untreated (T=0) or treated with tunicamycin (5 ug/mL) for 8h (T=8) before qRT-PCR was used to investigate mRNA expression levels of S1P, ATF6, and CREB3L2. β-actin was used as endogenous control, and mRNA expression was normalized to siCtrl T=0. Bar graphs show relative mRNA levels represented as mean ± SD, based on three independent knockdown experiments. * p value = 0.04, Unpaired T-test, two-tailed. **(B)** Cell survival after treatment with increasing concentration of tunicamycin. Measurements were done 72 h after drug treatment. Nonlinear regression was performed to determine the IC50 for the cell lines depleted of TRMT6/61A compared to the control. Regression line is marked as a solid line on the graph. IC50 *** p-value=0.0003.Unpaired T-test, two-tailed.

Cells were treated with 5 µg/mL tunicamycin for 8 hours to induce ER stress and subsequent UPR activation (26). Using qRT-PCR, mRNA expression levels of the Site-1 protease (S1P), activating transcription factor 6 (ATF6), and cyclic AMP-responsive element-binding protein 3-like protein 2 (CREB3L2) were investigated 8 hours after tunicamycin treatment and showed increased mRNA expression suggesting UPR activation (**Figure 7A**). S1P is involved in the cleavage and formation of the active forms of transcription factors ATF6 (ATF6^1-373^) and CREB3L2. In 5637 cells, significantly lower mRNA levels of CREB3L2 were detected in siTRMT6/TRMT61A samples after 8h tunicamycin treatment compared to the control. In the next set of experiments, we investigated whether reduction of TRMT6/TRMT61A affected the ability to tolerate cellular stress in 5637 and HT1197 cells. Following knockdown of TRMT6/TRMT61A, cells were treated with increasing concentrations of tunicamycin (0, 50, 100, 200, 400, 600 nM), followed by an assessment of cell survival using the PrestoBlue assay 72 h after drug treatment (**Figure 7B**). In 5637, depletion of TRMT6/61A decreased the tolerance for tunicamycin, reflected by a significantly lower IC50 for cells treated with siTRMT6/61A compared to the control. In HT1197, cell survival remained unchanged, and as previously observed, these cells were seemingly unaffected by even high dosages of the drug.

### 3.8 m1A mismatch rate is decreased by TRMT6/TRMT61A knockdown but not altered by tunicamycin treatment

**Figure 8:**
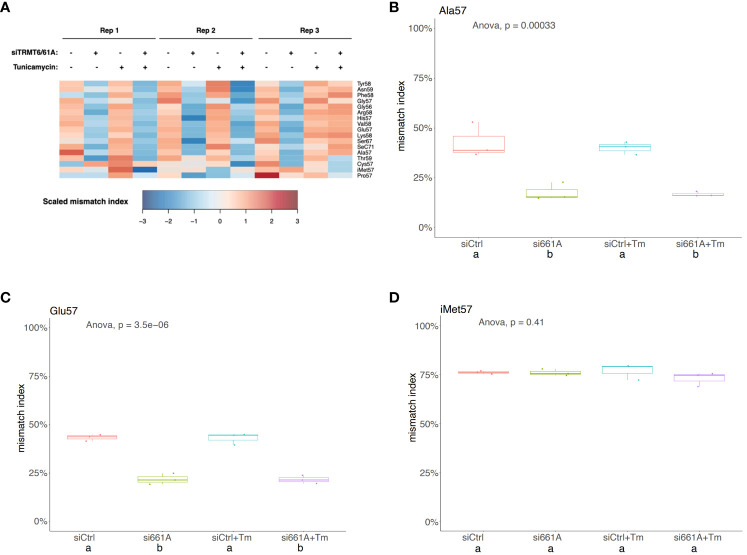
m1A mismatch rate is decreased by TRMT6/61A knock-down but not altered by tunicamycin treatment. **(A)** Heatmap representation of mismatch index of selected m1A sites from tRFs. Mismatch index is scaled across samples (rows). As expected, m1A mismatch rate is relatively decreased upon siTRMT6/61A. **(B-D)** Box plots showing m1A mismatch rate on tRFs from selected amino acid groups. Mismatch indexes from small RNA-seq are shown as box plots. One-way ANOVA was used to determine any global difference in mismatch indexes by the conditions (p value shown). *Post-hoc* Tukey test (confidence level 0.95) was used to examine pair-wise comparisons. Each pair with significant difference (p < 0.01) are labeled with “a” and “b”.

TGIRT-based small RNA-seq was performed to investigate whether depletion of TRMT6/TRMT61A altered expression levels of small RNAs in the 5637 cell line. No significant changes in small RNA expression in siTRMT6/TRMT61A compared to siCtrl were detected (**Supplementary Figures 2A, B**). Cells were treated with 5 µg/mL tunicamycin for 8 hours to induce ER stress and UPR activation. However, no significant changes in small RNA expression in siTRMT6/siTRMT61A treated with tunicamycin compared to siCtrl treated with tunicamycin were detected (**Supplementary Figures 2C, D**). Similar to our previous report, the m1A mismatch rate was relatively decreased in siTRMT6/TRMT61A samples across a range of tRFs (**Figure 8A**). On the other hand, tunicamycin treatment did not seem to have a significant effect on the tRF m1A modification status (**Figures 8B–D**).

## 4 Discussion

BLCA is a disease with high incidence and a contributor to mortality worldwide. Clinical management of the disease poses challenges due to a high level of heterogeneity and, thus, variable responses to treatments. Hence, there is a need for improved treatment of BLCA. The posttranscriptional RNA modification m1A is gaining increased attention as the aberrant expression of its regulatory proteins is associated with various cancers, including glioma (14), lung (27), and pancreatic (28, 29) cancer. High TRMT6 has also been reported in BLCA patients (16, 17), and elevated TRMT61A expression level in the 5637 BLCA cell line is suggested to play a central role in the pathogenesis of BLCA (16). Here, we characterized the expression levels of m1A methyltransferase complex TRMT6 and TRMT61A, as well as the m1A demethylases ALKBH1 and ALKBH3 in five BLCA cell lines of different BLCA grades and stages and in a control cell line derived from a healthy donor. The 5637 cell line showed elevated TRMT6 protein expression in contrast to most of the other BLCA cell lines, which had comparable protein expression levels as the control SV-HUC1. Apart from T24, all BLCA cell lines had upregulated levels of TRMT61A. However, the two subunits are both required for m1A methyltransferase activity (9). Thus, the collective m1A methyltransferase activity is upregulated in 5637 compared to the SV-HUC1 control. It also seems plausible to assume this to be the case for HT1376 and SW780, despite these cell lines having only modestly elevated TRMT6 expression compared to SV-HUC1.

m1A has been linked to various cellular processes, including cell proliferation, cell migration, and invasion, which are all central features of cancer cells. It has been shown that m1A in tRNAs can promote cell proliferation. In glioma, glioblastoma, and hepatocellular carcinoma, overexpression of TRMT6/TRMT61A promotes proliferation and malignant transformation (13–15, 30). In line with this, our results showed that the downregulation of the TRMT6/TRMT61A m1A methyltransferase complex reduced cell proliferation in both 5637 and HT1197 cell lines. The downregulation of TRMT6 was recently found to suppress glioma cell migration and invasion (14). In contrast, ALKBH3 promotes cancer cell invasion through m1A demethylation, causing tRNA destabilization (12). Here, cell tracking and subsequent cell displacement analysis of 5637 cells after TRMT6/TRMT61A knockdown revealed that the average cell displacement speed was significantly reduced compared to the control. Similarly, this trend was seen in wound-healing assays for the investigation of migration properties.

The UPR is an intracellular signaling pathway utilized by eukaryotic cells to restore endoplasmic reticulum (ER) homeostasis during stress-related conditions (31). The pro-survival UPR has been reported as an important factor in the progression of several cancers, including BLCA (32). In the T24 BLCA cell line, we linked TRMT6/TRMT61A activity of mediating m1A modification status on tRF-3s to a role in the maintenance of UPR homeostasis (17). In the current study, downregulation of TRMT6/TRMT61A and subsequent treatment with the UPR stress inducer tunicamycin showed a significantly lower level of mRNA expression of CREB3L2 and slightly reduced mRNA levels of S1P, which have been previously identified as targets for m1A-modified tRF3-mediated silencing (17). Although this requires further investigation, the results could suggest that the ATF6-branch of the UPR is affected by the TRMT6/TRMT61A-m1A-axis in the 5637 cell line. The HT1197 cell line did not seem to be affected by the same mechanism. Notably, a variable degree of Tm sensitivity has been detected across gastric cancer (GC) cell lines, wherein MDR GC cells were more sensitive to Tm than the parental cells, and the increased Tm sensitivity was suggested to correlate with basal ER stress levels (33).

If the UPR cannot restore the ER to normality, ER stress leads to cell dysfunction and, ultimately, cell death (34). Here, we wanted to investigate if depletion of TRMT6/TRMT61A combined with tunicamycin-mediated induction of UPR would influence BLCA cell survival. Upon downregulation of TRMT6/TRMT61A in 5637 but not HT1197 cells had a significantly reduced cell survival rate after treatment, even with low concentrations of tunicamycin. Moreover, TGIRT-based small RNA-seq of 5637 samples revealed that depletion of TRMT6/TRMT61A resulted in decreased m1A mismatch rate across a range of tRFs compared to siCtrl which was expected. However, tunicamycin treatment did not seem to influence the m1A mismatch index of these tRFs. Based on these findings, it seems like TRMT6/TRMT61A may be involved in desensitizing BLCA cells against cellular stress. This could offer an interesting opportunity to investigate if these cells with a compromised stress tolerance would be more susceptible to chemotherapeutic agents commonly used in BLCA treatment to mitigate the risk of recurrence, such as mitomycin C. Notably, further studies would include a TRMT6/TRMT61A knockout model system to give more insight into the role of the TRMT6/TRMT61A methyltransferase complex in BLCA.

BLCA is broadly categorized into NMIBC and MIBC, which comprise heterogeneous subtypes (2). In this study, the 5637 and HT1197 cell lines were used in our investigations, representing NMIBC and a metastatic BLCA, respectively. The two cell lines do not seem to respond similarly to the assays conducted after downregulating TRMT6/TRMT61A expression, which could reflect that the underlying mechanisms for BLCA pathogenesis differ in the two systems. It further raises the question of whether TRMT6/TRMT61A is linked to any grade or stage of BLCA development. Provided that the mutation rates in these cell lines have been characterized and found to differ (35), this should not be ruled out.

## Data availability statement

The raw data supporting the conclusions of this article will be made available by the authors, without undue reservation.

## Ethics statement

Ethical approval was not required for the studies on humans in accordance with the local legislation and institutional requirements because only commercially available established cell lines were used.

## Author contributions

IM: Conceptualization, Formal analysis, Investigation, Methodology, Validation, Writing – original draft, Writing – review & editing, Data curation, Software, Visualization. LL: Conceptualization, Data curation, Investigation, Methodology, Visualization, Writing – original draft. JR: Data curation, Formal analysis, Visualization, Writing – review & editing. FK: Data curation, Visualization, Writing – review & editing. AL: Data curation, Formal analysis, Visualization, Writing – review & editing. SB: Data curation, Formal analysis, Visualization, Writing – review & editing. AD: Data curation, Methodology, Project administration, Supervision, Writing – review & editing. ZS: Data curation, Formal analysis, Methodology, Supervision, Validation, Visualization, Writing – original draft, Writing – review & editing. AK: Conceptualization, Data curation, Formal analysis, Funding acquisition, Investigation, Methodology, Project administration, Resources, Supervision, Validation, Visualization, Writing – review & editing. RO: Conceptualization, Data curation, Formal analysis, Funding acquisition, Investigation, Methodology, Project administration, Resources, Supervision, Validation, Visualization, Writing – original draft, Writing – review & editing.
