# Supplementary material for: Depletion of the m1A writer TRMT6/TRMT61A reduces proliferation and resistance against cellular stress in bladder cancer
Source: Front Oncol. 2024 Jan 18;13:1334112. doi: 10.3389/fonc.2023.1334112 (PMC10830773; doi:10.3389/fonc.2023.1334112)
Supplement: Supplementary file 1 [file DataSheet_1.docx]

Supplementary Material

Article Title

Ida Monshaugen, Luisa Luna, Jayden Rhodes, Felicia Iselin Svensson Kristiansen, Anna Lång, Stig Ove Bøe, Anindya Dutta, Zhangli Su, Arne Klungland, Rune Ougland^*^

*** Correspondence:** Rune Ougland: runoug@vestreviken.no

**Supplementary Data**

# Supplementary Figures and Tables

For more information on Supplementary Material and for details on the different file types accepted, please see [here](https://www.frontiersin.org/guidelines/author-guidelines#supplementary-material).

## Supplementary Figures

**Table S1**: qRT-PCR primers used for qRT-PCR experiments.

| **Target** | **Sequence (5’-3’)** |
| --- | --- |
| TRMT61A forward | ACAGACATCGCCCTCATCAC |
| TRMT61A reverse | GATGATGGCGTGGGACACAG |
| TRMT6 forward | AAGGGGCCTAAAGAGAGAGGA |
| TRMT6 reverse | TAGCTACAATTAAACCATCTGCGT |
| ALKBH1 forward | TTCCTGAGGTATAAAGAAGCGACT |
| ALKBH1 reverse | GAGGAAACCCAGGTCAGAAGG |
| ALKBH3 forward | AGCCACGAGTGATTGACAGA |
| ALKBH3 reverse | TCACGTCAACAAAGCCAGGA |
| ATF6^1-373^ forward | GCCTTTATTGCTTCCAGCAG |
| ATF6^1-373^ reverse | TGAGACAGCAAAACCGTCTG |
| S1P forward | ACGCTTCACACGTTCGGATGAG |
| S1P reverse | TGACAGGTGGTCACTCCTCATG |
| CREB3L2 forward | ACCACACGCACTTCTCAGAAC |
| CREB3L2 reverse | GAGGAAAGGATCATTCAGGAGC |
| ACTB forward | ACAGAGCCTCGCCTT |
| ACTB reverse | CGCGGCGATATCATC |
| GAPDH forward | AATCAAGTGGGGCGATGC |
| GAPDH reverse | GCAGTTGGTGGTGCAGGA |


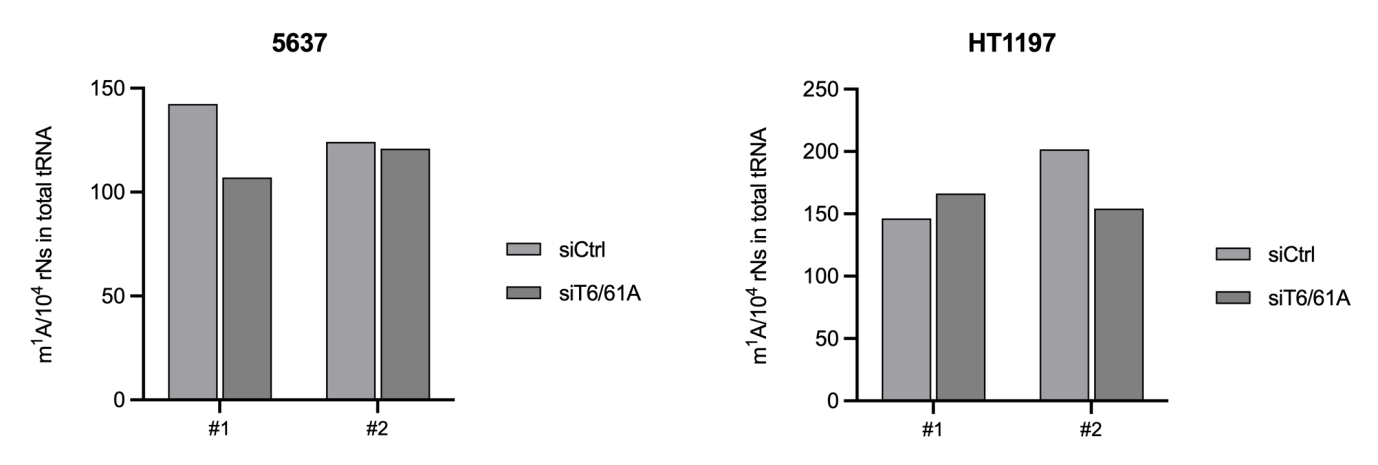


**Figure S1:** **Quantification of m1A modification in total tRNA by LC-MS/MS.** m^1^A total tRNA of 5637 and HT1197 was determined relative to unmodified nucleosides. The data represents two independent experiments per cell line.


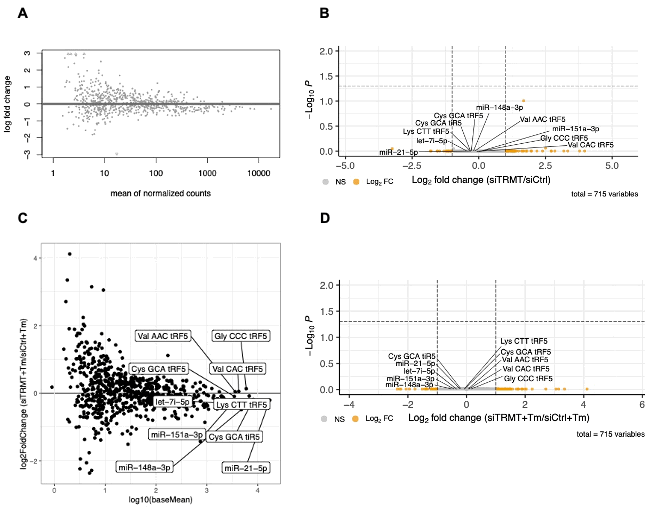


**Figure S2: No significant changes in small RNA expression after TRMT6/61A knock-down. (A, B)** No significant changes (adjusted p value < 0.05) in small RNA expression in siTRMT6/61A (n = 3) compared to siCtrl (n = 3). Top 10 expressed small RNAs are labeled. **(C, D**) No significant changes (adjusted p value < 0.05) in small RNA expression in siTRMT6/61A+Tm (n = 3) compared to siCtrl + Tm (n = 3). Top 10 expressed small RNAs are labeled.


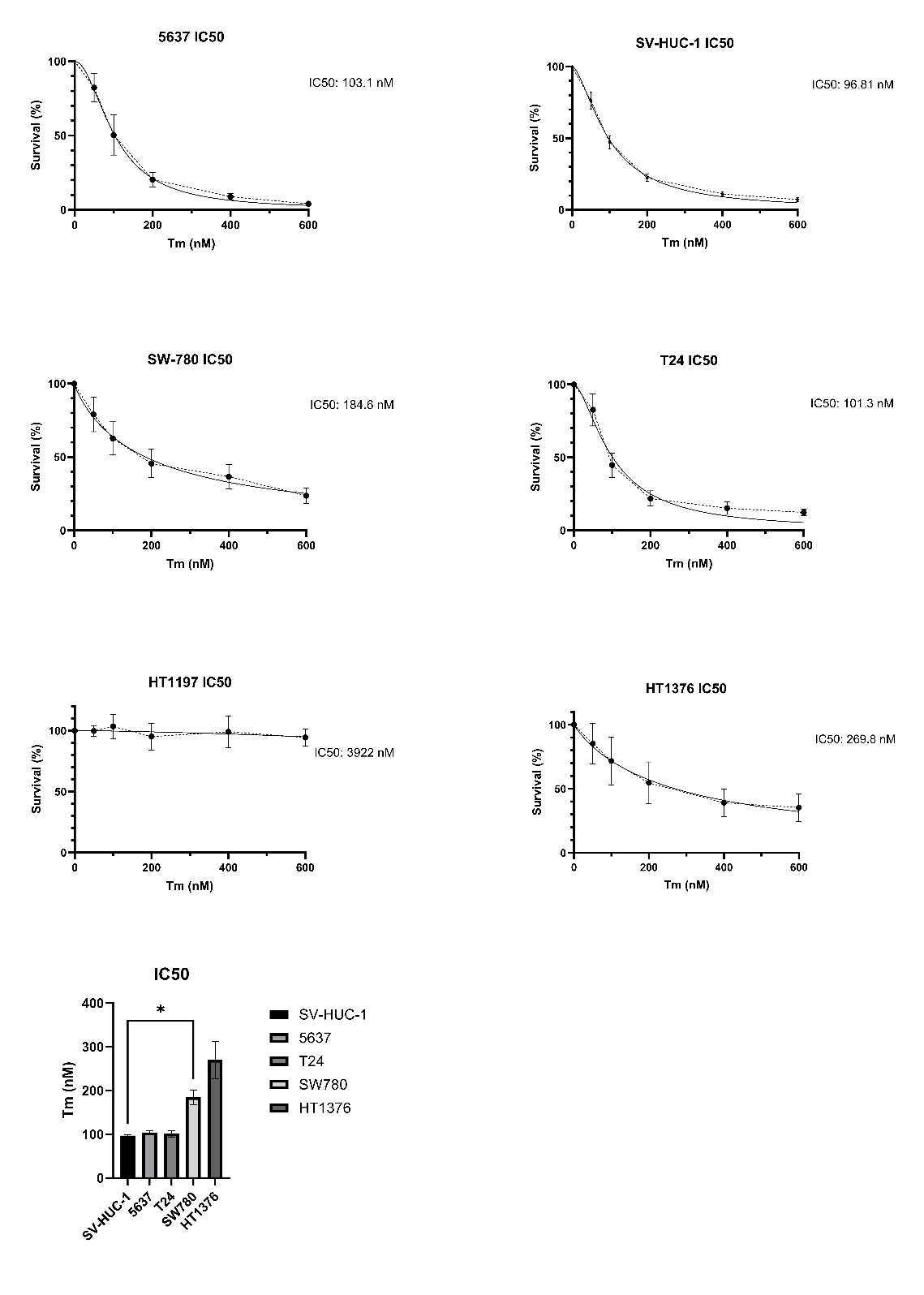


**Figure S3: Determination of IC50 for Tm in BLCA and control cell lines.** IC50 for Tm in the different cell lines was analyzed using the non-linear regression equation [inhibitor] vs. normalized response in GraphPad Prism 10.1.1. Each graph visualizes the data for each cell line with a solid line for the regression curve fit. The corresponding IC50 is annotated on the right. IC50 was compared against SV-HUC1 using a two-way Welch’s t-test. HT1197 was not included in the graph, as the IC50 regression model was not suitable for this dataset. Data is presented as mean ± SD.
